# Avian Influenza H9N2 Seroprevalence among Poultry Workers in Pune, India, 2010

**DOI:** 10.1371/journal.pone.0036374

**Published:** 2012-05-18

**Authors:** Shailesh D. Pawar, Babasaheb V. Tandale, Chandrashekhar G. Raut, Saurabh S. Parkhi, Tanaji D. Barde, Yogesh K. Gurav, Sadhana S. Kode, Akhilesh C. Mishra

**Affiliations:** National Institute of Virology, Microbial Containment Complex, Pashan, Pune, India; Duke-NUS Gradute Medical School, Singapore

## Abstract

Avian influenza (AI) H9N2 has been reported from poultry in India. A seroepidemiological study was undertaken among poultry workers to understand the prevalence of antibodies against AI H9N2 in Pune, Maharashtra, India. A total of 338 poultry workers were sampled. Serum samples were tested for presence of antibodies against AI H9N2 virus by hemagglutination inhibition (HI) and microneutralization (MN) assays. A total of 249 baseline sera from general population from Pune were tested for antibodies against AI H9N2 and were negative by HI assay using ≥40 cut-off antibody titre. Overall 21 subjects (21/338 = 6.2%) were positive for antibodies against AI H9N2 by either HI or MN assays using ≥40 cut-off antibody titre. A total of 4.7% and 3.8% poultry workers were positive for antibodies against AI H9N2 by HI and MN assay respectively using 40 as cut-off antibody titre. This is the first report of seroprevalence of antibodies against AI H9N2 among poultry workers in India.

## Introduction

Influenza viruses belong to the family *Orthomyxoviridae*. Influenza viruses have segmented, single-stranded negative-sense RNA viruses and are classified into type A, B and C. They are divided into subtypes based on the serogrouping of 16 hemagglutinin (HA) and 9 neuraminidase (NA) genes. At least 103 of the possible 144 type A influenza virus HA-NA combinations have been found in wild birds.

Avian influenza (AI) H9N2 virus is a low-pathogenic virus with widespread distribution in poultry in Asia [1]. In Asia, AI H9N2 viruses have been regularly isolated from ducks [2]. However, during the later half of the last decade, H9N2 viruses have caused disease outbreaks in terrestrial poultry in many parts of the world [3]. It has been reported that AI H9N2 viruses have acquired receptor binding characteristics typical of human strains, increasing the potential for reassortment in both human and pig respiratory tracts [4], [5]. In immunosuppressed chickens, the H9N2 virus causes severe respiratory tract infections with high mortality in young chicks and severe decline in egg production in laying chickens, which results in economic loss. This virus persists in chicks and spreads to non-affected flocks through fecal-oral route without showing much of severe clinical signs [6]. Crossing the species barrier to mammals highlights the pandemic potential of AI H9N2 viruses.

AI H9N2 virus was isolated for the first time from humans in Hong Kong in 1999 [7], which led to the fears that H9N2 virus could become a potential pandemic candidate apart from H5N1 virus. In 2003, human cases of H9N2 virus were recorded in Hong Kong although no death was reported [8], [9]. Studies have shown that avian H9N2 virus isolated from chickens is closely related to human H9N2 isolate from Hong Kong [10]. A human case of AI H9N2 has been recently reported from Bangladesh a neighbouring country of India [1]. These events prompted a series of sero-epidemiological studies worldwide, which showed seroprevalence of AI H9N2 in the range of 1–6% in different risk groups [11]–[13].

AI H9N2 virus circulation within live bird markets in India has been reported [14], [15]. The seroprevalence of AI H9N2 has also been reported in emus (*Dromaius novaehollandiae*) from India [16]. In this scenario it is essential to conduct animal-human interface studies in India. The present study reports findings of seroepidemiological study of AI H9N2 among poultry workers in Pune, Maharashtra, India.

## Materials and Methods

### Subject selection, risk factors, ethics, consent and sample collection

The poultry shops and farms were identified in and around Pune city for contacting the poultry workers for invitation to participate in the study. Samples were collected from wet poultry markets and poultry farms. Type of birds sold in poultry markets and farms were mostly chickens. Chickens were either broiler or backyard chickens. Approximately 50 birds were kept in each shop in poultry markets while poultry farms size ranged from 1000 to 10,000 chickens. These markets and farms were in urban, semi-urban or rural areas.

The written informed consent was obtained from individual study participants. The informed consent form included the information about the study, its relevance, utility and study procedures including risks and benefits. The “National Institute of Virology Ethical Committee for Research on Human Subjects” approved the study. The study participants were interviewed and enquired for the pre-existing co-morbid diseases/conditions or illnesses in the recent past (6-months), current/routine nature of work, and any other work assignments of similar or related nature.

**Figure 1:**
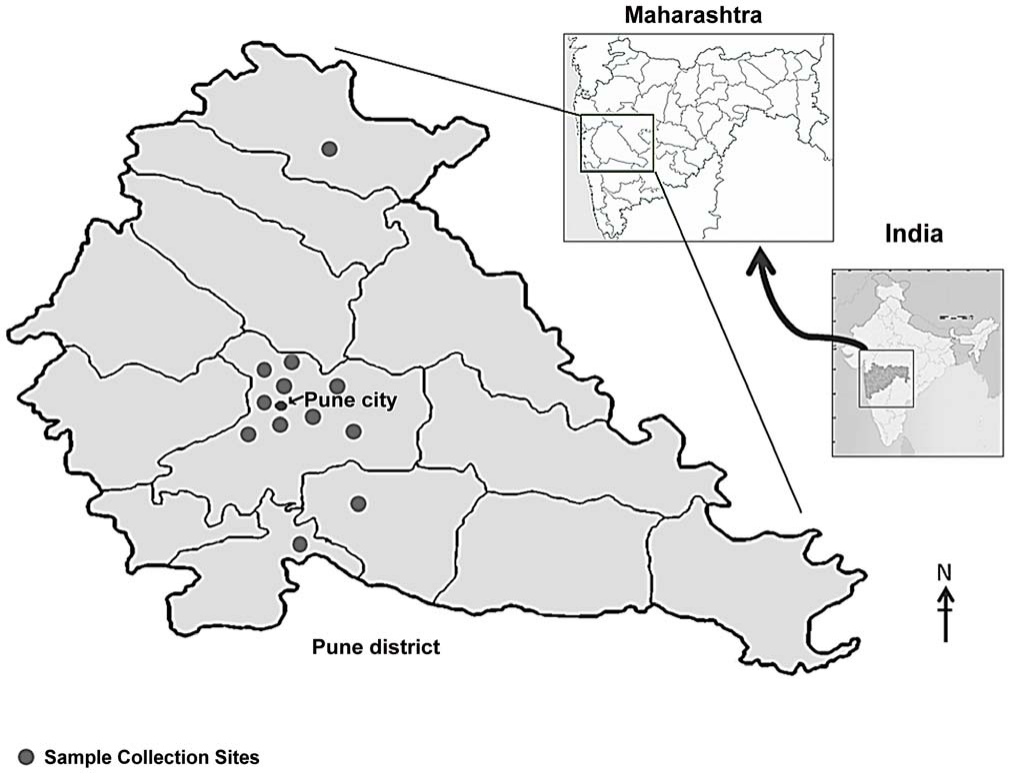
Sample collection sites in Pune city and district.

The poultry workers were the individuals involved in handling, transport, cleaning and slaughter of poultry. A person showing presence of antibodies against AI H9N2 by either hemagglutination inhibition (HI) or microneutralization (MN) assay was considered as seropositive. As there is no published report available on number of poultry workers working in Pune, attempts were made to represent poultry worker population (Figure 1). Therefore an assumption of 500 poultry workers was made for sample size calculations for this pilot study. An assumption of 5% antibody prevalence against AI H9N2 virus was made based on the published reports of similar studies performed outside India [11]–[13]. The sample size calculations were performed using SSCPS version1001 (SPSS) [17]. A total of 338 poultry workers were enrolled in the study during July–December 2010. The estimated sample size was 239 assuming 5% prevalence, 95% confidence, and precision of 0.02% by 2-sided test with finite population correction with population size of 500 for Pune city. The samples were collected from 30 locations in Pune city and adjoining area. 2–3 ml of blood samples were collected by venepuncture and serum was separated and stored at −20°C until tested. A total of 249 baseline sera collected from general population from Pune were used to determine baseline antibody level against AI H9N2 virus.

### Viruses used in the study

For influenza antibody detection by both HI and MN assays, influenza A/Chicken/India/NIV/99321/09 (H9N2) virus isolated at the National Institute of Virology (NIV), India was used. Virus was propagated in the allantoic cavities of 10-day-old embryonated chicken eggs. The allantoic fluid was harvested after 48 hours post-infection. The virus was titrated by hemagglutination (HA) assay using 0.5% turkey red blood cells (RBCs).

### Hemagglutination Inhibition (HI) assay

For HI assay, all serum samples were treated with receptor destroying enzyme (Denka Seiken Co. Ltd., Chuo-ku, Tokyo, Japan) to remove non-specific inhibitors. Serum samples showing the presence of agglutinins were treated with horse and turkey RBCs to remove non-specific agglutinins. The presence of non-specific agglutinins was evident by the hemagglutination of RBCs by the sera. Such serum samples were adsorbed with turkey RBCs. One volume of packed RBCs were mixed with 20 volumes of RDE treated serum and incubated at 37°C for 1 hour, centrifuged at 1200 rpm for 10 minutes. Adsorbed serum was carefully removed without disturbing packed cells and used in the HI assay. HI assays were performed using 0.5% turkey RBCs [18]. Since reference cut-off values of both HI and MN assays for AI H9N2 serosurveillance in humans are not available, results of HI and MN assays were reported using ≥40, ≥80 and ≥160 antibody cut-off titres.

### Microneutralization (MN) assay

MN assays were performed with Madin-Darby canine kidney (MDCK) cells obtained from the Centres for Disease Control and Prevention (CDC), Atlanta, USA. The cells were used for a maximum of 25 passages and maintained in Dulbecco's modified Eagle's medium (DMEM) containing 10% fetal bovine serum (Gibco®, Grand Island, NY, USA), 2 mM L-glutamine and the antibiotics penicillin and streptomycin. The 50% tissue culture infectious dose (TCID_50_) of H9N2 virus was determined by titration in MDCK cells. The TCID_50_ was calculated according to Reed et al. [19].

Briefly, 1/2-log dilutions of virus were carried out in 100 µl of DMEM containing 1% bovine serum albumin, TPCK trypsin (2 µg/ml) and antibiotics (virus diluent) in high-binding 96-well polystyrene immunoassay plates (Nunclon™ Delta surface, Nunc, Denmark). Freshly trypsinized MDCK cells were adjusted to 1.5×10^5^/ml in virus diluents and 100 µl was added to each well. The plates were covered and incubated for 18–22 hours at 37°C and 5% CO_2_. The monolayers were washed with phosphate-buffered saline (PBS) and fixed in cold 80% acetone in PBS for 10 minutes. The presence of viral nucleoprotein (NP) was detected by Enzyme linked immunosorbent assay (ELISA) using influenza A-specific anti-nucleoprotein monoclonal antibodies (Millipore, Temecula, CA, USA) [20]. Wells having an absorbance reading greater than 3 standard deviations above the mean absorbance of wells containing only MDCK cells were scored positive for virus growth.

Sera were heat-inactivated for 30 minutes at 56°C and two-fold serial dilutions were performed in 96-well polystyrene immunoassay plates. The diluted sera were mixed with an equal volume of virus diluent containing influenza virus at 100 TCID_50_/50 µl. Four control wells of virus plus virus diluent (VC) or virus diluent alone (CC) were included on each plate. After a 1-hour incubation at 37°C in a 5% CO_2_ humidified atmosphere, 100 µl of MDCK cells at 1.5×10^5^/ml was added to each well. The plates were incubated for 18–22 hours at 37°C and 5% CO_2_. The monolayers were washed with PBS and fixed in cold 80% acetone for 10 minutes. An ELISA was performed as described above.

## Results

The study subjects were mostly males (87.28%). Median age of poultry worker was 25 years (n = 330). There were no co-morbid disease conditions. Also, vaccination against seasonal and pandemic influenza H1N1 2009 virus was not reported by any participant. Influenza like illness was not reported by study subjects during the last six months from the day of blood collection.

TCID_50_ of AI H9N2 was 10^5.25^. All 249 baseline sera were negative using ≥40 antibody cut-off titre by HI assay. Results are reported with ≥40, ≥80 and ≥160 antibody cut-off titres. Overall 21 subjects (21/338 = 6.2%) were positive by either HI or MN assays using ≥40 cut-off antibody titre. Eight subjects (8/21 = 38%) were common positives in both HI and MN assays (Table 1). A total of 4.7%, 1.1% and 0.6% subjects were positive for antibodies against AI H9N2 using ≥40, ≥80 and ≥160 antibody cut-off titres respectively using HI assay. Similarly, 3.8%, 1.5% and 0.3% samples were positive using ≥40, ≥80 and ≥160 antibody cut-off titres respectively, using MN assay.

**Table 1:** Hemagglutination and microneutralization assay results.

Subjects	Antibody titres		
	≥40	≥80	≥160
	HI MN	HI MN	HI MN
**Poultry workers ** ***(n = 338)***	16 13	04 05	02 01
**% Positivity**	4.7 3.8	1.1 1.5	0.6 0.3

## Discussion

Occupational exposure to infected poultry has been an important factor in AI virus transmission to humans [21]. In the present study, the presence of antibodies against AI H9N2 in poultry workers suggests the probable subclinical infection. Enquiries did not indicate respiratory illness in the recent past. The mean age group of individuals positive for antibodies against AI H9N2 was 28.6 years, indicating susceptibility of young adults to AI H9N2. This could be due more number of younger subjects in the study. Sporadic cases of human infection with AI H9N2 usually present with relatively mild symptoms in humans [8], [22], [23]. Human infections with AI H9N2 have been reported in China, Hong Kong and Vietnam [8], [11], [22]–[24]. A human case of confirmed H9N2 infection was reported recently in Bangladesh, where patient was involved in handling of dead chicken, washing and cutting the meat in house prior to onset of influenza like illness [1]. Therefore in the current scenario of circulation of AI H9N2 in India, AI surveillance in poultry workers and especially cullers is urgently required in India to trace AI virus infections from poultry.

Phylogenetic analysis has shown that human H9N2 isolates between 1997 and 2009 belong to G1 and Y280 lineages [25]. The AI H9N2 virus isolated from India belonged to the G1-like lineage of H9N2 [26]. AI H9N2 virus remains a pandemic concern for humans as this virus shows a high level of genetic plasticity, exhibiting extensive evolution. Experiments with reassortant strain have shown that favourable mutations replacing other amino acids by leucine at amino acid position 226 shows affinity towards human receptors (2,6 sialic acid) and are able to infect ferrets and transmit the virus efficiently [27], [28]. Therefore episodes of H9N2 virus into humans may create opportunities for reassortment with co-circulating human viruses and an opportunity for the genesis of new influenza strains with pandemic potential. Thus, AI surveillance and virus characterization are necessary to understand the characteristics of low pathogenic AI viruses.

As reference antibody cut-off values of HI or MN assays are not available for detection of antibodies in humans, results were reported with cut-off tires ≥40 as well as ≥80 and ≥160 antibody titres by both HI and MN assays. India reported outbreaks of highly pathogenic avian influenza (HPAI) H5N1 in poultry since 2006. The evidence of AI (H9N2) in poultry market may provide the opportunity for human infections and the possibility of reassortment with the existing poultry AI viruses including HPAI H5N1 virus. Regular cleaning and disinfection of wet poultry markets have been found to be helpful in preventing chain of transmission of AI viruses in Indonesia [29]. Such attempts would also help India to curtail spread of AI viruses in wet poultry markets and exposure to humans. The limitation of this study is that generalization cannot be done due to fewer numbers of samples. Therefore, this study does not empower comparison of prevalence rate at the markets versus farms or correlation of prevalence within sites versus among sites. The present pilot study showed low prevalence of antibodies against AI H9N2 virus, which is comparable with reported studies from South-East Asia. Further virological and serological studies in poultry workers are urgently required in India to monitor human infections.
